# Case report: Intramuscular ketamine or intranasal esketamine as a treatment in four patients with major depressive disorder and comorbid anorexia nervosa

**DOI:** 10.3389/fpsyt.2023.1181447

**Published:** 2023-05-15

**Authors:** Johanna Louise Keeler, Janet Treasure, Hubertus Himmerich, Madeline Brendle, Claire Moore, Reid Robison

**Affiliations:** ^1^Department of Psychological Medicine, King's College London, Institute of Psychiatry, Psychology and Neuroscience, London, United Kingdom; ^2^South London and Maudsley NHS Foundation Trust, Bethlem Royal Hospital, Beckenham, Kent, United Kingdom; ^3^Department of Pharmacotherapy, University of Utah College of Pharmacy, Salt Lake City, UT, United States; ^4^Numinus Wellness, Draper, UT, United States; ^5^Department of Psychiatry, University of Utah School of Medicine, Salt Lake City, UT, United States

**Keywords:** anorexia nervosa, depression, esketamine, intranasal, intramuscular, ketamine, case report

## Abstract

**Introduction:**

A comorbid diagnosis of a depressive disorder is a negative prognostic factor for individuals with AN, and novel treatments are needed to target depressive symptoms in this population. One emerging promising treatment for depressive disorders is ketamine, although there is less research investigating the use of ketamine for alleviating depression in people with AN.

**Case report:**

This study reports on four patients with a lifetime diagnosis of AN and a comorbid diagnosis of major depressive disorder who received either intramuscular ketamine (*n* = 2) or intranasal esketamine (*n* = 2) treatment from a private psychiatric clinic. Depressive symptomatology (PHQ-9) was measured prior to (es)ketamine administration on every dosing session and adverse effects were recorded during and after dosing. All patients reported a subjective decrease in depression, although only those administered intranasal esketamine showed a reduction in PHQ-9 depression scores over time. Number of doses ranged from 3 to 23. All patients tolerated treatment well and no serious adverse effects emerged, however nausea/vomiting was experienced by one patient on one dosing session. Weight remained stable in all cases, although notably across all patients, weight at the beginning of treatment was within a “healthy” range.

**Discussion:**

These findings suggest that (es)ketamine may reduce depressive symptoms in people with major depressive disorder and a comorbid diagnosis of AN. Future feasibility and pilot trials are warranted in order to elicit robust data on efficacy, acceptability, safety and tolerability.

## 1. Introduction

Anorexia nervosa (AN) is a serious and persistent restrictive eating disorder, which has the highest mortality of any psychiatric disorder, attributable to both the physical sequalae of low weight and high rates of suicide (1–3). Depressive disorders are a common comorbidity with AN, present in ~50% of patients (4). Depression is a poor prognosis marker for patients, with one study finding that a comorbid diagnosis of an affective disorder resulted in a six-fold higher likelihood of remaining unwell with AN after a 15-year follow-up (5). Early improvements in depressive symptomatology have been found to be a positive prognostic factor for recovery from AN (6).

Patients with AN have reported that engaging in disordered eating is a coping strategy to deal with, and ameliorate, negative emotions (7). However, low mood may increase despondency in patients and consequently result in poor treatment engagement and efficacy. Treatment options for AN are largely centered around clinical management of weight-related risk and psychological intervention. There are currently no licensed pharmacological options, and standard antidepressants tend to be ineffective in this population (8). There is an urgent demand for novel treatments in this population, particularly those that may alleviate low mood.

Ketamine is an n-methyl-D-aspartate (NMDA) receptor antagonist that in low doses stimulates glutamate transmission (9). It was traditionally used as an anesthetic agent, although more recently has been utilized in the treatment of psychiatric disorders. In particular, ketamine has shown efficacy for treatment-resistant depression due to its rapid anti-depressant properties that have been found to occur within 24 h of dosing and last for up to 1 week (10). Numerous meta-analyses have demonstrated the efficacy of ketamine for treating unipolar and bipolar depression in increasing response and decreasing remission rates, as well as suicidal ideation (10–16). There are several administration routes for ketamine, each with differing levels of bioavailability. In order of bioavailability, these are: intravenous (100%), intramuscular (90–95%), subcutaneous (90–95%), intranasal (30–50%), sublingual (20–30%), transdermal (10–50%) and oral (10–20%) (17). There are two enantiomers, the S(-) and R(-) forms, which when are combined in a 1:1 racemic mixture are described as ketamine [(R,S)-ketamine]. Esketamine refers to the S(+) enantiomer of ketamine. Meta-analytic evidence may indicate the superiority of the racemic formulation of ketamine over esketamine in treating both unipolar and bipolar depression (12).

Despite transdiagnostic similarities, there is a paucity of research investigating ketamine in AN populations in comparison to other psychiatric conditions such as depression and anxiety disorders (18). There are several existing studies examining the use of ketamine for treating eating disorders. The first investigation of ketamine as a treatment for eating disorders was conducted by Mills et al. (19), where 15 patients were administered 20 mg/h of intravenous ketamine for 10 hours in order to treat compulsive behaviors. Of the 15, nine showed a sustained decrease in compulsive behaviors. More recently, several other case studies have detailed improvements in depression and eating disorder symptomatology in patients with bulimia nervosa (20), AN (21) and protracted eating disorders more generally (22), using various formulations of ketamine and treatment programmers.

Most studies combined ketamine administration with psychotherapy and/or dietary intervention [e.g. an eating disorder recovery programme; (23), or a ketogenic diet; (21, 24)], and it is unclear how ketamine as a solitary intervention impacts outcomes in patients with eating disorders. Moreover, there are few studies investigating changes in depression and anxiety in patients with AN in particular.

Symptoms of depression tend to be particularly resistant to treatment in patients with AN, which may be a function of low weight and the associated reduction in gray matter of the brain of ~3–9% (25). Improvements in depressive symptoms may be an early indicator of change in patients with AN and ketamine theoretically may be effective even in patients with low body weight, where other antidepressants generally are not. It has been purported that a neuroprogressive feature of AN is reduced neuroplasticity (26, 27), which ketamine, through various pathways (e.g. effects on synaptogenesis), may target (26). Overall, there is currently a high level of interest in this area but crucially a need for more evidence for the use of ketamine for patients with AN (26).

Moreover, depression is common in patients with AN and is often treatment resistant. There is a need for more evidence investigating changes in depressive symptomatology in patients with comorbid AN and depression. Therefore, the present study sought to examine patterns of depression symptomatology over time in four patients with a lifetime diagnosis of AN and major depressive disorder (MDD) who were treated with intramuscular ketamine or intranasal esketamine. Furthermore, safety and tolerability information in this patient group is scarce in the literature. Therefore, secondary aims were to investigate data on adverse effects in this patient group.

## 2. Method

### 2.1. Participants

Participants were four female patients who visited a private outpatient psychiatric clinic between 2018 and 2021. Participants were approached retrospectively and were included in the study if they had a primary diagnosis of MDD and a secondary diagnosis of anorexia nervosa, and were treated with two or more doses of intramuscular ketamine or intranasal esketamine. Diagnoses were made by a psychiatrist according to the Diagnostic and Statistical Manual of Mental Disorders 5th Edition (28). Demographic and clinical characteristics of the included patients are displayed in Table 1.

**Table 1:** Demographic and clinical characteristics of patients.

**Patient**	**Age at first session**	**Ethnicity**	**Type of ED**	**Comorbidities**	**Route of administration and ketamine formulation**	**Maximum dosage (mg)**	**No. Doses**	**Medication usage at baseline**
A	36	Caucasian	AN-BP	TRD, GAD	Intramuscular Ketamine	200	3	Aripiprazole; Duloxetine; Gabapentin; Lamotrigine; Mirtazapine; Modafinil; Prazosin; Temazepam
B	23	Caucasian	AN	MDD, GAD, PTSD	Intramuscular Ketamine	90	7	Duloxetine; Lorazepam; Lamotrigine; Gabapentin; Ativan
C	19	Caucasian	AN	TRD	Intranasal esketamine	84	23 (N.B. PHQ-9 recorded for 19)	Quetiapine; Fluoxetine
D	19	Caucasian	Historical AN and BN	TRD	Intranasal esketamine	84	10	Alprazolam; Desvenlafaxine succinate; Gabapentin; Metoclopramide; Naltrexone; Quetiapine; Risperidone

AN, anorexia nervosa; AN-BP, AN binge-purge subtype; BN, bulimia nervosa; ED, eating disorder; GAD, generalized anxiety disorder; MDD, major depressive disorder; PTSD, post-traumatic stress disorder.

Ethical approval for the present study was obtained from the King's College London Research Ethics Committee and retrospective informed consent was obtained in writing from all patients.

### 2.2. Investigations and treatment

Patients were referred to a private outpatient psychiatric clinic, where they received an initial psychiatric assessment by a psychiatrist (R.R.). All referrals were primarily for the treatment of depressive symptomatology. Prior to ketamine/esketamine treatment, patients were screened for medical fitness by a clinician (i.e., a medical doctor, nurse practitioner or physician assistant). Following this, the same clinician was responsible for the ongoing management of the patient's ketamine/esketamine treatment plan, including the dosage, session frequency and number of sessions. The session frequency and duration of treatment was decided collaboratively between the clinician and the patient, and three-monthly reviews were conducted in order to assess ongoing suitability and necessity for ketamine/esketamine treatment.

Before each ketamine/esketamine session, patients discussed their goals and intentions for treatment with their clinician. Vital signs were assessed prior to administration and antiemetic medication (e.g., ondansetron 8 mg) was offered. In order to assess depressive symptomatology, the Patient Health Questionnaire-9 [PHQ-9; (29)] was administered immediately before each dosing session. The PHQ-9 is a 9-item questionnaire, widely used for the measurement of the severity and extent of depressive symptomatology. Items are rated on a 4-point Likert scale, ranging from 0 (“Not at all”) to 3 (“Nearly every day”) and pertain to symptoms over the previous 2 weeks. A total depression score is calculated as a sum of responses to all items in the respective questionnaires. Scores of 0–4 are indicative of no depressive symptoms, 5–9 of mild depression, 10–14 of moderate depression, 15–19 of moderately severe depression and 20–27 of severe depression.

Patients received ketamine/esketamine whilst reclined in a chair, in a quiet room with dimmed lights. As per standard protocol, patients self-administered esketamine under clinician supervision, starting with a dosage of 56 mg and titrating up to a maximum dosage of 84 mg where appropriate. Intramuscular ketamine was administered by a clinician, with the dosage typically between 0.5 and 0.7 mg/kg, titrated up as tolerated and required per each visit (by ~0.1–0.2 mg/kg), but up to a maximum of 200 mg. In order to facilitate an “inward journey”, patients were supplied with an eye-mask, headphones and music, which consisted of relaxing non-lyric music curated by clinic staff. During treatment, which lasted ~2 h, patients' blood pressure and heart rate was monitored by the patient's clinician, who was responsible for recording and responding to emergent adverse events. The following adverse event data were recorded: hallucinations/delirium, nausea/vomiting, unstable vitals, and “other adverse events”.

### 2.3. Analysis of data

Descriptive statistics were used to present within-subject changes in depressive symptomatology across the dosing period for the four participants. Time series diagrams were used to illustrate session-by-session symptom scores at successive time intervals.

## 3. Outcome and follow-up

**Figure 1:**
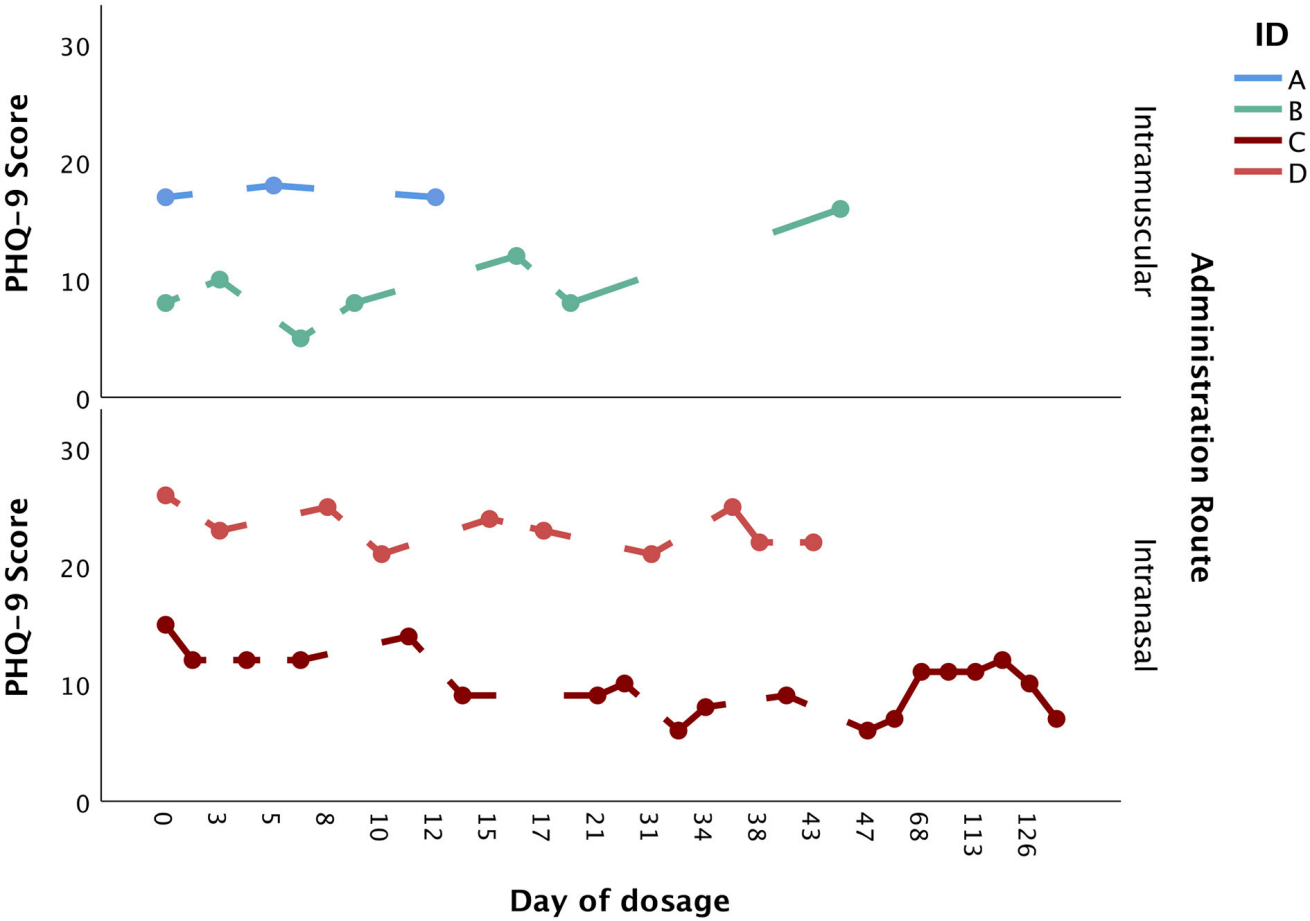
Changes in PHQ-9 score over the dosage period, stratified by administration route (intramuscular vs. intranasal).

A total of four patients, who were seen between 2018 and 2021, were included in the present paper. All patients were adult Caucasian females (≥18 years of age) with a primary diagnosis of MDD. All patients were receiving concurrent antidepressant medication. Two patients (patients A and B) received dosages of intramuscular ketamine, and two patients (patients C and D) received dosages of intranasal esketamine. The dosage period was variable, ranging from 12 to 149 days. Similarly, the total number of doses was variable, ranging from 3 to 23 doses. Figure 1 provides a visual depiction of changes in PHQ-9 scores over the dosage period, stratified by route of administration.

### 3.1. Case descriptions

#### 3.1.1. Intramuscular ketamine administration

**Patient A** was a 36-year-old Caucasian woman with a current diagnosis of AN (binge-purge subtype), MDD (treatment-resistant) and generalized anxiety disorder. She had three intramuscular ketamine doses over 12 days, with the dosage reduced from 200 mg in the first dosage, to 100 mg in the second dosage and 75mg in the final dosage. Although the PHQ-9 score remained stable over the three doses (baseline: 17, final dose: 17), the patient subjectively reported noticing an improvement throughout treatment, albeit noted this was transient. Moreover, the patient noted improvements in suicidal ideation. Following ketamine treatment, her diagnosis changed to stable (at goal) AN. Her weight at the first dosage was 68.5 kg (body mass index; BMI = 22.9 kg/m^2^) and at the final dosage was 67.6 kg (BMI = 22.6 kg/m^2^), indicating that her weight remained stable over the total dosage period.

**Patient B** was a 23-year-old Caucasian woman with a diagnosis of AN, MDD, generalized anxiety disorder and post-traumatic stress disorder. She received a total of seven intramuscular ketamine doses over a period of 44 days, with the initial dosage of 30 mg titrated up to 90 mg. Whilst the PHQ-9 score increased from 8 at first dose, to 16 at seventh dose, the patient reported subjectively noticing a decrease in depressive symptoms and thus wished to proceed with ketamine treatment. Two psychiatric follow-ups were conducted after this treatment period, at two- and five-months, which saw a worsening of depression. The patient reported engaging with psychological therapies for her eating disorder during this time and felt motivated to eat, although wished to recommence ketamine treatment to improve her mood as subjectively viewed it as helpful in the past. The patient recommenced ketamine treatment (1 dose of 100 mg recorded, ~10 months after the initial ketamine treatments). At two long-term psychiatric follow-ups at 1- and 6-months following this single intramuscular ketamine dosage, the patient reported some persisting anxiety and depression although felt that the symptoms were manageable. No adverse effects were reported throughout the treatment period. At the first and last dosage, her weight was 46.3 kg (BMI = 18.5 kg/m^2^), indicating that her weight remained stable over the dosage period.

#### 3.1.2. Intranasal esketamine administration

**Patient C** was a 19-year-old Caucasian woman with a diagnosis of AN and MDD (recurrent and treatment-resistant), who received a total of 23 intranasal esketamine doses (albeit the PHQ-9 score was recorded for only 19) over a period of 149 days. The dosage was titrated from 56 mg in the first two doses to 84 mg in the subsequent doses. The baseline PHQ-9 score was 15, which lowered to 7 by the end of treatment. Subjectively the patient reported seeing an initial decrease in depression, which was largely sustained in the following dosing sessions. No adverse effects were reported throughout the treatment period. At the first dosage, her weight was 51.1 kg (BMI = 21.3 kg/m^2^), which increased to 54.5 kg by the last dosage (BMI = 22.7 kg/m^2^).

**Patient D** was a 19-year-old Caucasian woman with a historical diagnosis of AN and BN, and a current diagnosis of MDD (treatment-resistant). Patient D received 10 intranasal esketamine doses over a period of 43 days. The dose was titrated from 56 mg in the first two doses, to 84 mg in the third dose, which was then titrated down to 56 mg in the subsequent seven doses. Her starting PHQ-9 score was 26, which decreased to 22. Subjectively, the patient reported a decrease in depression that was sustained in the following dosing sessions, although notably the depression severity was still clinically significant at the end of treatment. Overall, the intranasal esketamine was tolerated well, albeit nausea/vomiting was reported on seventh dose. At the first dosage her weight was 50.7 kg (BMI = 19.1 kg/m^2^), which increased to 53.4 kg at the final dosage (BMI = 20.1 kg/m^2^).

## 4. Discussion

The initial aim of this study was to assess changes in depressive symptomatology in four patients with a diagnosis of AN and MDD treated with intramuscular ketamine or intranasal esketamine. Additionally, this study aimed to assess the presence of adverse effects in this patient group. All patients reported a subjective decrease in depression, although scores derived from the PHQ-9 indicated a reduction in depression only in patients treated with intranasal esketamine. Weight remained stable in the two patients treated with intramuscular ketamine and increased in the two patients treated with intranasal esketamine. Overall, both forms of ketamine were tolerated well, with only one patient reporting nausea and/or vomiting on one occasion. Importantly, all patients had a BMI above 18.5 kg/m^2^, and one of the patients (patient D) treated with intranasal esketamine had a lifetime diagnosis of AN rather than an acute diagnosis. These results indicate that (es)ketamine may reduce depressive symptoms in people with a comorbid diagnosis of AN, which may improve functioning and quality of life.

The mechanism of action of ketamine is thought to be related to the modulation of glutamate transmission *via* NMDA and α-amino3-hydroxy-5-methyl-4-isoxazolepropionic acid (AMPA) receptors (30). It is thought to have downstream effects in activating brain-derived neurotrophic factor and mechanistic target of rapamycin (mTOR) signaling pathways, potentiating synaptic plasticity. Additionally, 5-hydroxytryptamine (5-HT)_1A_ and 5-HT_1B_ receptor agonism may be important for the antidepressant effects of ketamine, as an effect of NMDA receptor inhibition and AMPA receptor activation (31). These potential mechanisms of action are relevant for both MDD and AN where neuroplasticity and serotonergic signaling may be impaired (26). Several case studies and case series have provided preliminary evidence for the use of ketamine in patients with MDD and comorbid AN (19, 21, 24, 32). However, the empirical basis is currently meager and there has been less of a focus on depressive symptoms as a proximal indicator of change in patients, despite other research that investigates novel biologically-based therapeutics in AN (e.g., repetitive transcranial magnetic stimulation to the left dorsolateral prefrontal cortex) suggesting early changes in depression as an indicator of treatment efficacy (33, 34). The current case series provides some support for use of (es)ketamine to target depressive symptoms in people with a diagnosis of MDD and AN.

There was no overall effect of ketamine treatment on BMI in this patient group, although all patients included were of a reasonable BMI. Indeed, most studies investigating ketamine as a treatment for AN have not used patients that are underweight, which may be related to safety concerns in this population. This is a major limitation within the field as a contrast to the clinical reality; the majority of individuals who are acutely unwell with AN are underweight, and ketamine treatment may be a particular indication for patients with protracted illness and long-term low weight where other treatment options have failed (26). There is a vital need for well-designed randomized controlled trials in this area, to assess the feasibility and efficacy of using ketamine in underweight patients with AN. Trials should pay attention to particular contraindications or risks that may be associated with this population, such as the presence of liver enzyme, cardiac or hepatic abnormalities [see Keeler et al. (26) for a further discussion of side effects and safety concerns when treating AN with ketamine]. Importantly, outcomes that may be proximal markers of change in this population, such as depressive symptoms and quality of life, might be indicators of efficacy for such trials.

The standard of ketamine administration for depression has been a (0.1–0.75 mg/kg) intravenous infusion over 40 mins, administered and supervised by an anesthetist. However, there is emerging evidence for the efficacy of alternative administration routes, such as intranasal and intramuscular, such as those used in the present study. These differ by bioavailability. Intramuscular has ~90–95% bioavailability and intranasal has ~30–50% bioavailability (17). There is meta-analytic evidence for the efficacy of both routes of administration (35). Both routes have a number of advantages over intravenous administration. For example, the administration does not require infusion equipment. However, there may be slightly more discomfort with intramuscular administration in comparison to intranasal. Ultimately, the optimal dosage and route of administration may differ depending on individual preference and clinical response, which should be established collaboratively between patient and clinician. For example, it is possible that the dissociative side effects of ketamine may be less tolerable for patients with high levels of anxiety, who may benefit from forms of ketamine that are less likely to produce psychotomimetic or dissociative effects [e.g. oral or intranasal; (36)]. Studies would furthermore benefit from investigating routes of administration that have not yet been explored in AN populations, but have shown efficacy in affective and anxiety disorder populations, such as orally administered ketamine (37, 38).

### 4.1. Strengths and limitations

This case series provided preliminary evidence for the efficacy and tolerability of a novel treatment, low-dose (es)ketamine, administered through two different routes, in a patient population that thus far has received little attention in the ketamine literature. Comorbid depression is a negative prognostic factor for patients with AN (5), and treatments targeting depression in this population are urgently needed. However, owing mainly to the study design, there are several limitations to the current manuscript.

This investigation used pre-existing data obtained from a private psychiatric clinic administering (es)ketamine to patients with treatment-resistant depression as a primary diagnosis. Therefore, there was no measure of eating disorder psychopathology available for further contextualization of the efficacy of (es)ketamine in this patient group, which is a limitation. However, as aforementioned, changes in eating disorder psychopathology may be a distal marker of change as opposed to changes in depressive symptoms, which may be more proximal, in such treatments designed for treatment-resistant cases (33, 34). Other tools for the measurement of depressive symptoms may be more comprehensive than the PHQ-9, which in the current study appeared to be discrepant with the patients' own subjective experience of changes in their depressive symptomatology. The PHQ-9 consists of only nine items, three of which are closely related to features of AN and are likely to be scored highly (i.e., those pertaining to poor appetite and sleep, and hyperactivity). Measurement tools with more items [e.g. the Beck Depression Inventory; (39)] or subscales to isolate these overlapping symptoms [such as the Montgomery-Asberg Depression Rating Scale (40)] may be more suitable for measuring depressive symptoms in this population. Importantly, studies should also aim to integrate Patient Reported Outcome Measures (PROMs) as well as other important indicators of improvement, such as quality of life, anxiety symptoms and social integration.

The case study design has limitations in terms of heterogeneity across dosing protocol and patient characteristics (e.g. psychiatric comorbidity), which was tailored to the individual presentation of the patients (e.g. number of doses ranging from 3 to 22), as well as the small sample size. Finally, the patients included in this study were not underweight, meaning that the efficacy, safety and tolerability data yielded from this study are not applicable to the majority of AN cases where patients are underweight. This remains an issue in the field, where no well-controlled studies have been conducted in order to explore the potential of this important treatment in underweight individuals, where safety concerns may be amplified.

Further studies should examine the effect of (es)ketamine on core aspects of AN psychopathology, using measures of eating disorder psychopathology, quality of life and other outcomes identified by individuals with lived experience of AN, preferably within the context of a well-designed randomized controlled trial. In order for this to be possible, initial studies utilizing a Phase 1 feasibility study design will be necessary, to confirm and exemplify the feasibility, tolerability and acceptability of (es)ketamine as a treatment for patients with MDD and comorbid AN, particularly in acute cases of AN where BMI is low.

## 5. Conclusion

This case series included four patients with MDD and a comorbid lifetime diagnosis of AN who were treated with intranasal esketamine or intramuscular ketamine. All cases reported a reduction in depressive symptoms, although only those treated with esketamine showed a reduction in PHQ-9-rated depressive symptoms. Weight remained stable over the dosing period in all cases, although notably all cases had a BMI above 18.5 kg/m^2^ when commencing treatment. Both intranasal and intramuscular (es)ketamine were tolerated well, with a low incidence of adverse effects and nausea/vomiting occurring only on one occasion in one patient. This case series provides tentative preliminary evidence for the suitability of (es)ketamine treatment in reducing depressive symptoms in patients with MDD and a comorbid diagnosis of AN. There is a marked need for well-designed feasibility and pilot studies investigating (es)ketamine as a treatment for AN, with long-term follow-ups in order to assess proximal and distal indicators of change.

## Data availability statement

The original contributions presented in the study are included in the article/supplementary material, further inquiries can be directed to the corresponding author.

## Ethics statement

The studies involving human participants were reviewed and approved by the Research Ethics Office at King's College London. The patients/participants provided their written informed consent to participate in this study. Written informed consent was obtained from the individual(s) for the publication of any potentially identifiable images or data included in this article. Written informed consent was obtained from the participant/patient(s) for the publication of this case report.

## Author contributions

JLK: conceptualization, formal analysis, investigation, data curation, writing—original draft, writing—review and editing, visualization, project administration, and funding acquisition. JT and HH: conceptualization, writing—review and editing, and supervision. MB and CM: investigation, data curation, writing—review and editing, and project administration. RR: conceptualization, methodology, investigation, resources, data curation, writing—review and editing, supervision, project administration, and funding acquisition. All authors contributed to the article and approved the submitted version.
